# Characterization and miRNA Profiling of Extracellular Vesicles from Human Osteoarthritic Subchondral Bone Multipotential Stromal Cells (MSCs)

**DOI:** 10.1155/2021/7232773

**Published:** 2021-10-09

**Authors:** Clara Sanjurjo-Rodríguez, Rachel E. Crossland, Monica Reis, Hemant Pandit, Xiao-nong Wang, Elena Jones

**Affiliations:** ^1^Translational and Clinical Research Institute, Newcastle University, Newcastle upon Tyne, NE2 4HH, UK; ^2^Leeds Institute of Rheumatic and Musculoskeletal Medicine, University of Leeds, Leeds LS9 7TF, UK; ^3^Cell Therapy and Regenerative Medicine Group, Physiotherapy, Medicine and Biomedical Sciences Department, Universidade da Coruña, A Coruña 15006, Spain; ^4^Department of Pediatrics, Harvard Medical School, Boston, MA 02115, USA; ^5^Leeds Teaching Hospitals NHS Trust, Leeds LS7 4SA, UK

## Abstract

Osteoarthritis (OA) is a heterogeneous disease in which the cross-talk between the cells from different tissues within the joint is affected as the disease progresses. Extracellular vesicles (EVs) are known to have a crucial role in cell-cell communication by means of cargo transfer. Subchondral bone (SB) resident cells and its microenvironment are increasingly recognised to have a major role in OA pathogenesis. The aim of this study was to investigate the EV production from OA SB mesenchymal stromal cells (MSCs) and their possible influence on OA chondrocytes. Small EVs were isolated from OA-MSCs, characterized and cocultured with chondrocytes for viability and gene expression analysis, and compared to small EVs from MSCs of healthy donors (H-EVs). OA-EVs enhanced viability of chondrocytes and the expression of chondrogenesis-related genes, although the effect was marginally lower compared to that of the H-EVs. miRNA profiling followed by unsupervised hierarchical clustering analysis revealed distinct microRNA sets in OA-EVs as compared to their parental MSCs or H-EVs. Pathway analysis of OA-EV miRNAs showed the enrichment of miRNAs implicated in chondrogenesis, stem cells, or other pathways related to cartilage and OA. In conclusion, OA SB MSCs were capable of producing EVs that could support chondrocyte viability and chondrogenic gene expression and contained microRNAs implicated in chondrogenesis support. These EVs could therefore mediate the cross-talk between the SB and cartilage in OA potentially modulating chondrocyte viability and endogenous cartilage regeneration.

## 1. Introduction

Osteoarthritis (OA) is a heterogeneous disease that affects synovial joints, which starts from injury or as a molecular alteration and results in structural changes, such as loss of cartilage, bone sclerosis or osteophyte formation, pain, and other clinical symptoms [1]. OA is one of the most prevalent joint diseases and frequently causes disability, with high incidence worldwide due to population aging [2–4].

Early OA changes in articular cartilage include regional proteoglycan loss, chondrocyte clustering, collagen disorganization, and tissue fibrillation [5]. While the catabolic/anabolic imbalance in bone is associated to overloading microdamages, in cartilage it seems to be associated with persistent, low-grade inflammation [6]. It begins in the synovial tissue where it is characterized by cytokine release and immune cell infiltration and is commonly associated with pain in OA [7, 8]. With OA progression, subchondral bone (SB) gains more influence on OA chondrocytes as osteochondral junction becomes more porous [5, 6, 9–13]. Bone-targeting treatments have a positive effect on OA chondrocytes [6] while cocultures of OA subchondral bone osteoblasts [10] or osteoclasts [14] with OA chondrocytes have a negative effect on chondrocytes' viability or anabolic activity. The bone-cartilage crosstalk is therefore emerging as a novel therapeutic target for OA [13, 15].

In OA, different types of SB cells enter normally the nonvascular calcified cartilage through the cracks and fissures or along the newly formed vessels, all contributing to cartilage destruction “from below” [12, 16–18]. These SB cells include osteo/chondroclasts that dissolve the calcified cartilage matrix [18, 19], neurons that exacerbate joint pain [13, 20, 21], and multipotential stromal cells (MSCs) that may be involved in both, cartilage formation and cartilage degradation [12, 18]. Besides the cells themselves, SB-cartilage communication in OA may be mediated via soluble proteins [22], for example, VEGF or IL6, which are increased in damaged SB areas in OA [23], and or via extracellular vesicles (EVs); the influence of which on OA chondrocyte homeostasis remains poorly understood [24].

EVs are nonreplicative bilipid-layered particles that are released naturally from most of the cells, carrying peptides, miRNAs, lipids, and other molecules in their cargo, which can be transmitted to other cells. They can be broadly subcategorised based on their biogenesis, size, and markers into exosomes, microvesicles, and apoptotic bodies [25–27]. EVs have a crucial role in cell-to-cell communication, allowing the cargo transfer through endocytic internalization or direct fusion to provoke a biological response [28–31]. Recently, studies of EVs in joint diseases and their therapeutic use started to rise [24, 32–35], but these studies are primarily focused on EVs derived from osteoarthritic synovial fluid rather than SB [36, 37].

The aim of this study was to investigate EV production from SB MSCs, the cells previously shown to contribute to disease pathogenesis in OA [18, 38, 39]. We hypothesised that SB MSC-EVs may have an influence on OA chondrocytes' viability and their anabolic gene expression and that they contain specific sets of miRNAs that are different from their parental MSCs.

## 2. Materials and Methods

### 2.1. Patients and Cells

Ethical approval for cartilage and MSC osteoarthritic sample collection was obtained from the NRES Committees Yorkshire & The Humber—South Yorkshire (14/YH/0087) and NRES Committees North East—Newcastle & North Tyneside 1 (14/NE/1212). From this collection, 11 cartilage samples (6 females and 5 males with median age 74 years, range 55–83) and 5 subchondral bone samples (3 females and 2 males with median age 73 years, range 64–83) were collected from total knee arthroplasty.

Healthy MSC collection was obtained from surplus material remaining in 10 bone marrow-processing bags used for hematopoietic stem cell transplantation (NRES Committee North East—Newcastle & North Tyneside 2, 14/NE/1136). Patients were 7 males and 3 females with the median age of 14 years (range 4–44).

### 2.2. Isolation and Culture of OA Chondrocytes

Articular cartilage was harvested from tibiofemoral surfaces, and chondrocytes were isolated as previously described [18]. Briefly, the cartilage was harvested and minced using a scalpel and digested with collagenase overnight. Chondrocytes were expanded in a high-glucose Dulbecco's Modified Eagle Medium (DMEM; MilliporeSigma, USA) supplemented with glutamine, 10% fetal bovine serum (FBS; ThermoFisher Scientific, USA), and 1% penicillin/streptomycin (P/S; MilliporeSigma, USA). Media were changed twice a week, and subculture was performed when chondrocytes reached 80% confluence, and passaged to passage 2 (p2).

### 2.3. Isolation and Culture of MSCs

Osteoarthritic MSCs were obtained from the subchondral bone of medial femoral condyles after removal of cartilage, as previously described [18]. Medial condyles were chosen as they commonly display a more prominent OA phenotype compared to lateral condyles [18, 40]. Bone was weighted and mechanically minced into small fragments with a rongeur and digested with collagenase, as previously described [18]. MSC cultures were established in StemMACs™ MSC expansion media (Miltenyi Biotec, Germany) then transferred to DMEM supplemented with 5% human platelet lysate (PL; PLTMax, MilliporeSigma), 100 IU/ml penicillin, 100ug/ml streptomycin, 2,500 IU/ml heparin and 2 mM L-glutamine (all from MilliporeSigma) (5%PL/DMEM).

Healthy control MSCs were cultured from surplus cells (wash-outs) of hematopoietic stem cell transplantation bags, as previously described [41]. Briefly, bone marrow mononuclear cells (MNCs) were isolated by density gradient centrifugation and cultured in 5%PL/DMEM.

Both types of MSCs were previously characterized [18, 41] (Supplementary Figures 1 and 2) according to the criteria set by the International Society of Cellular Therapy (ISCT) [42] and expanded in 5%PL/DMEM to passage 3 before changing the media to EV-depleted 5%PL/DMEM, for EV isolation. EV-depleted MSC media were prepared by 18-hour ultracentrifugation of 10%PL/DMEM at 100,000 × *g* followed by 1 : 1 dilution in DMEM.

### 2.4. EV Isolation from OA and Control MSCs

EVs were isolated from both osteoarthritic (OA-EVs) and control healthy (H-EVs) p3 MSCs, as previously described [41]. In brief, when the cells reached 50% confluence, they were washed twice with phosphate buffered saline (PBS, MilliporeSigma) and cultured in a 5%PL/DMEM-EV-depleted medium, for a further duration of 48 hours prior to harvesting the conditioned media and MSC-EV isolation. In addition to collecting the conditioned medium, the number of MSCs in the flask was counted and an aliquot of cells was frozen as pellets in a QIAzol lysis reagent (Qiagen, Germany) for RNA isolation. To obtain MSC-EVs, the conditioned medium was centrifuged at 400 × *g* for 5 min, 2,000 × *g* for 20 min at 4°C, then transferred to ultracentrifuge tubes (Beckman Coulter, USA) and centrifuged again sequentially at 10,000 × *g* for 45 min and at 100,000 × *g* for 90 min at 4°C, using a 45Ti rotor (Beckman Coulter) in a Optima XE-90 ultracentrifuge (Beckman Coulter). The MSC-EVs pellet was washed in PBS then resuspended in approximately 200-300 *μ*l sterile PBS and stored at −80°C.

### 2.5. MSC-EV Characterization

MSC-EV characterization was performed by electron microscopy (TEM), flow cytometry, and nanoparticle tracking analysis (NTA) [41].

For TEM, 5 *μ*l of MSC-EVs was adsorbed for 30 s onto a carbon-coated, glow-discharged grid. Excess liquid was removed and samples were stained with 1% uranyl acetate (Agar Scientific, UK). Excess uranyl acetate solution was removed, and the MSC-EV-loaded grids were dried then examined using a Hitachi HT7800 transmission electron microscope. Digital images were collected using an Emsis Xarosa camera with Radius software.

EV surface markers CD63, CD9, and CD81 were analyzed by flow cytometry following coating of 4 *μ*m aldehyde/sulfate latex beads (ThermoFisher Scientific) with 10 *μ*l of MSC-EVs suspension. The reaction was stopped by incubation with 1 M glycine (MilliporeSigma), and the MSC-EV-bead complex was washed twice with PBS then incubated with mouse anti-human PE CD63 (clone H5C6), PerCPCy5.5 CD9 (clone M-L13), and APC CD81 (clone JS-81) antibodies or corresponding isotype controls (all from BD Biosciences, USA). Following further washes, cells were resuspended in particle-free PBS (ThermoFisher Scientific) and data was acquired using a BD FACS Canto II cytometer (BD Biosciences) and analyzed with FlowJo 10.0 software (Tree Star Inc., USA).

For NTA, MSC-EV pellets were diluted with sterile particle-free PBS and analyzed using Nanosight LM10 (Malvern Panalytical Ltd, UK), as described by the manufacturer's protocol. Three 60 s measurements of the particle size and concentration were measured for each sample. The acquired data was processed using NTA 2.3 software (Malvern Panalytical Ltd).

### 2.6. ATP-Based Viability Assessment of Chondrocytes following Coculture with MSC-EVs

The CellTiter-Glo 2.0 Luminiscent Cell Viability Assay (Promega, UK) measures total ATP levels produced by metabolically active cells. For this assay, EV-depleted chondrocyte media was first prepared by 18-hour ultracentrifugation of DMEM/Nutrient Mixture F-12 (DMEM:F12, ThermoFisher Scientific) containing 10% FBS (DMEM:F12/10%FBS) at 100,000 x g and optimal conditions (5 × 10^3^ chondrocytes/well, 24-hour treatment duration) established by seeding different cell concentrations and determining the ATP levels.

To determine the effect of MSC-EVs (both OA- and H-EVs) on chondrocytes' ATP levels, EVs from 6 × 10^4^ MSCs were added into 300 *μ*l of EV-depleted DMEM:F12/10%FBS containing 1.5 × 10^4^ chondrocytes (MSC to chondrocyte ratio of 4 : 1 [43], and 100 *μ*l of the mix (containing 5 × 10^3^ chondrocytes) was seeded in triplicate wells of the same 96-well Nunclon delta plates. As a control, 5 × 10^3^ chondrocytes without MSC-EVs were plated in triplicate wells containing 100 *μ*l of the same EV-depleted DMEM:F12/10%FBS media. A second dose of EVs from 6 × 10^4^ MSCs was added on day 3, and luminescence measurements were performed on day 5. For this, 100 *μ*l of equilibrated CellTiter-Glo 2.0 reagent was added to the wells and luminescence was measured 10 min afterwards, using the Spark multimode microplate reader (Tecan, Switzerland) and the Sparkcontrol method editor software (Tecan). In these experiments, OA-EVs and H-EVs were from 3 and 6 MSC donors, respectively.

### 2.7. Three-Dimensional (3D) Pellet Coculture of MSC-EVs and OA Chondrocytes

To characterize whether MSC-EVs (both OA- and H-EVs) have an effect on chondrocytes' expression of genes implicated in the extracellular matrix (ECM) metabolism, EVs from 6 × 10^6^ OA or control MSCs were added to 6 × 10^5^ chondrocytes resuspended in EV-depleted DMEM:F12/10%FBS media (MSC-EVs to chondrocytes ratio 10 : 1). Afterwards, chondrocytes were separated into 3 different tubes and centrifuged at 450 × *g* for 10 min to create 3D pellets, for each treatment. In addition, 2 × 10^5^ chondrocytes were pelleted in EV-depleted DMEM:F12/10%FBS media without MSC EVs and used as negative controls (No EVs). After 2 and 7 days, EV-depleted media was half-changed and cell pellets were taken for RNA isolation. In these experiments, OA-EVs and H-EVs were from 2 different donors each, and the chondrocyte cultures (*n* = 3) were not donor-matched to OA-MSCs.

Longer-term effect of OA and healthy MSC-EVs on chondrocyte gene expression in the pellet culture was tested using a single-donor chondrocyte culture and MSC to chondrocyte ratio of 4 : 1, with half-change medium every 3-4 days.

### 2.8. RNA Isolation from OA and Healthy MSC-EVs, Parental MSCs, and MSC-EV-Treated Chondrocytes

For isolation of RNA from EVs (60 *μ*l of the EVs suspension in PBS), total Exosome RNA and Protein Isolation kit (ThermoFisher Scientific) were used following the manufacturer's instructions. The RNA concentration from EVs was assessed using the Bioanalyzer 2100 and the RNA 6000 pico kit (Agilent Technologies, USA).

RNA from parental MSCs (from which EVs were produced from) and from chondrocyte pellets was obtained using the miRNeasy Mini kit (Qiagen) following the manufacturer's instructions. The RNA was quantified using the Nanodrop spectrophotometer (ThermoFisher Scientific).

### 2.9. miRNA Profiling from OA and Healthy MSC-EVs

miRNA profiling was carried out using the nCounter® Human v3.0 miRNA Expression Assay Kit (NanoString Technologies), based on miRBase v21, from total RNA obtained from OA-EVs and H-EVs as well as parental MSCs (the MSCs from which the respective EVs were obtained from). The code set incorporated 799 mature microRNAs and included 6 positive controls, 8 negative controls, 6 ligation controls, 5 mRNA housekeeping controls (*ACTB, B2M, GAPDH, RPL19* and *RPLP0*) and 5 spike-in controls. miRtag ligation, miRNA CodeSet Hybridization and post-hybridization were performed following the manufacturer's instructions. The resulting miRNA expression profiles were analyzed using the nSolver software V4 (NanoString Technologies). Samples were normalized to the geometric mean of the Top100 miRNAs taking into account background thresholding and positive control normalization (geometric mean). Fold change (FC) expression differences between groups were calculated using nSolver v2.5 (NanoString Technologies) ratio data, based on normalized count data. Further analysis was performed using a pipeline designed by Newcastle University, Haematological Sciences Department. This integrated a number of R (R project) statistical packages in the R programming language; *p* values between two groups were generated using a two-tailed *t*-test. Analysis on miRNA targets was performed using mirWalk [44], mirPath from DIANA Tools [45], Reactome [46] and miRDIP [47], utilizing default parameters for Human or *Homo sapiens*.

### 2.10. Quantitative Real Time PCR

Gene expression was assessed in 2, 7, and 21 day-cultured OA and healthy MSC-EV-treated pellets and control chondrocyte pellets. Total RNA was isolated and reverse transcribed with the High-Capacity cDNA Reverse Transcription kit (Applied Biosystems). qRT-PCR was performed using the Taqman assays (ThermoFisher Scientific; Table 1) and Fast Advanced Master Mix (Applied Biosystems, ThermoFisher Scientific) in a QuantStudio 3 Real-Time PCR system (Applied Biosystems). Genes for the analysis were selected based on the literature evidence of their involvement in the chondrogenesis and chondrocyte catabolic and anabolic activity [12, 48] and are shown in Table 1.

## 3. Results

### 3.1. EV Characterization

**Figure 1:**
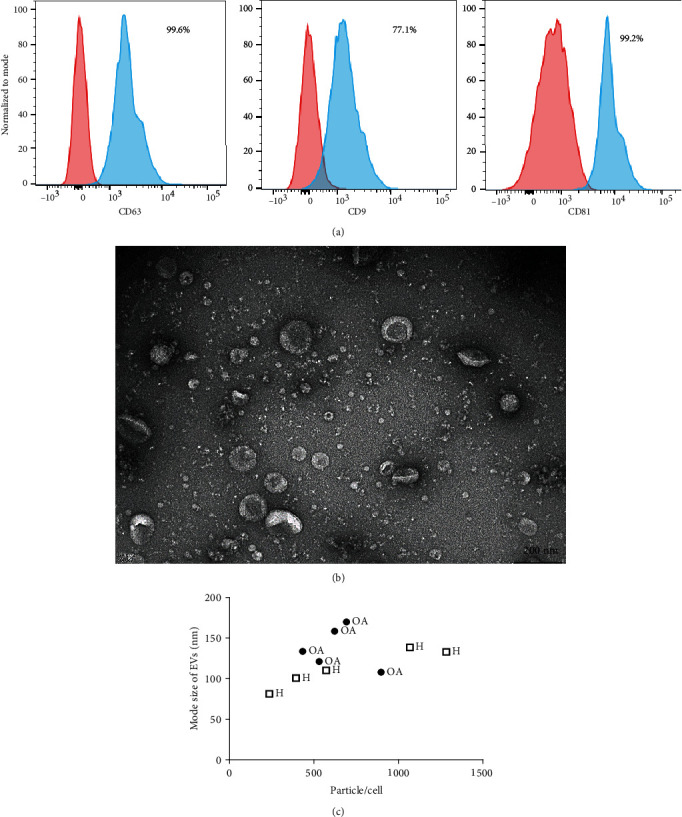
OA-MSC-EV characterization. (a) Flow cytometry of CD63, CD9, and CD81 surface markers (representative OA-EV sample). Red and blue histograms illustrate isotype controls and stained samples, respectively. (b) Transmission Electron Microscopy showing the cup-shape morphology of EVs (representative OA-EV sample, scale bar 200 nm). (c) Scatter plot showing size and number of particles/cell analysis of OA and control H-EVs, assessed by nanoparticle tracking analysis.

EVs isolated from OA-MSCs (*n* = 5) showed similar basic characteristics (Figure 1) than previously shown for control healthy MSCs [41]. Expression of different markers of small EVs [25] in OA-EV preparations was first tested using flow cytometry (Figure 1(a)). CD63 (95.9% ± 7.4%) and CD81 (90.1% ± 13.0%) were the highest expressed markers followed by CD9 (66.1% ± 23.8%) indicating presence of small EVs (Figure 1(a)). The morphology of OA-EVs observed using TEM was a typical cup-shape (Figure 1(b)). The concentration of particles and the size of the EVs was measured using NTA (Figure 1(c), Table 2). The mode of the OA-EV size ranged between 107.9-169.8 nm (Table 2) not significantly different to H-EVs (81.3-132.9), and consistent with the modal size previously reported for control H-EVs [41]. The mean concentration of particles per ml in OA-EV preparations was 1.38 × 10^11^ ± 2.07 × 10^10^ and is similar to that of the H-EVs (1.12 × 10^11^ ± 8.50 × 10^9^) (Table 2). As cell number was counted after OA and healthy MSC-EV media collection, no differences (*p* > 0.05) were found in the mean number of particles obtained per cell from either OA or control MSCs: 634 ± 176 and 709 ± 447, respectively. Neither difference was observed when comparing mean EV size between healthy and OA-MSC-EVs (*p* > 0.05): 112.7 ± 23.50 nm and 138.2 ± 25.68 nm, respectively, but OA-EVs seemed less variable than H-EVs (Figure 1(c)). These data indicated that OA-MSCs had a similar capacity to produce EVs with a comparable modal size and particle concentration as control healthy MSCs.

### 3.2. The Effects of OA and Healthy MSC-EVs on Chondrocyte Viability

**Figure 2:**
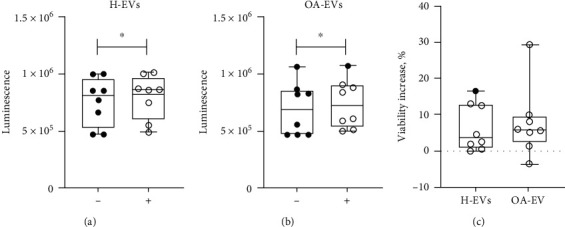
The effect of EVs on chondrocyte viability in CellTitter-Glo assay. (a) Viability of chondrocytes measured in luminescence units when treated with H-EVs (+), compared with untreated OA chondrocytes (-). (b) Viability of chondrocytes when treated with EVs from OA-MSCs (+), compared with untreated chondrocytes (-). (c) Percentage increase in viability above the untreated controls between H-EVs and OA-EVs. Horizontal bars show medians, and whiskers represent min to max. ∗*p* value < 0.05. Paired *t*-test analysis.

An ATP-based viability assessment was performed following coculture of OA chondrocytes with OA- or H-EVs. When comparing untreated chondrocytes with those treated with control H-EVs, an average 6.27% increase in chondrocyte viability was found (*p* value = 0.020) (Figure 2(a)). OA-EV treated chondrocytes also showed increased viability compared to untreated chondrocytes (average 5.92%, *p* value = 0.042) (Figure 2(b)). However, the difference in the percentage increase of viability between OA and H-EVs failed to reach statistical significance (*p* value = 0.768) (Figure 2(c)).

### 3.3. The Effects of OA and Healthy MSC-EVs on Chondrocyte Gene Expression

**Figure 3:**
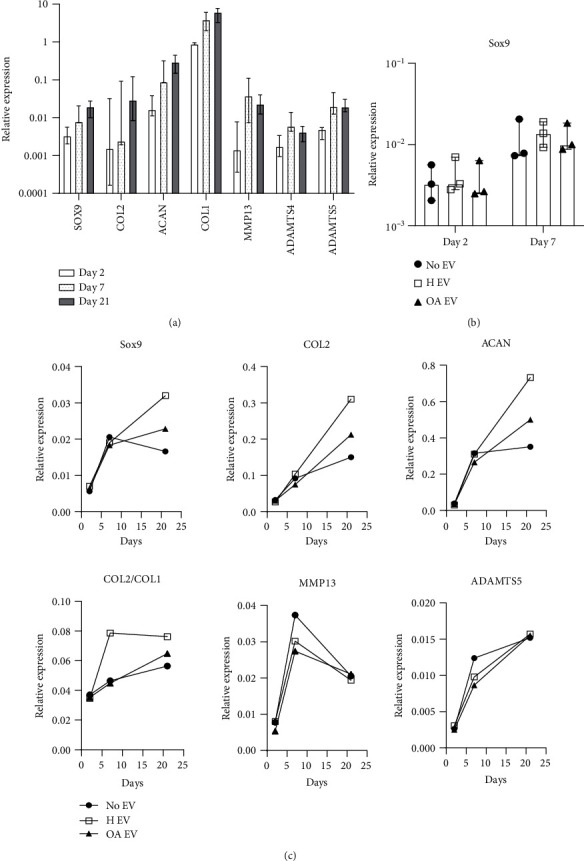
Gene expression of characteristic cartilage catabolic and anabolic genes measured in (a) untreated chondrocytes (no EVs) cultured for 2, 7, and 21 days. Horizontal bars show medians, and whiskers represent interquartile range. (b) *SOX9* expression in untreated and H-EV or OA-EV treated chondrocytes after 2 and 7 days. Symbols represent individual donor-derived chondrocyte cultures. (c) Chondrogenic gene expression in untreated (no EVs) and treated (H-EVs and OA-EVs) chondrocytes after 2, 7, and 21 days.

In these experiments, pellet culture of chondrocytes and EVs was used to facilitate EV uptake by chondrocytes, as reported previously [49]. The media used in these experiments did not contain any chondrogenic inducers because the latter could ‘mask' potentially smaller effects by EVs. To confirm that pellet culture environment was sufficient to induce chondrogenic gene up-regulation in chondrocytes, and to investigate how it affected the expression of the selected catabolic and anabolic genes, a time-course experiment in untreated pelleted chondrocytes from 4 donors was performed (Figure 3(a)).

As expected, gradual increases in chondrogenesis markers *SOX9*, *COL2* and *ACAN*, as well as *COL1*, were seen from day 2 onwards confirming that pellet culture in the absence of chondrogenic inducers was sufficient to monitor the effect of EVs on chondrogenic gene up-regulation. The up-regulation of other transcripts (*MMP13*, *ADAMTS4* and *ADAMTS5*) did not similarly continue beyond day 7 (Figure 3(a)).

An average 18% up-regulation of *SOX9* transcript compared to untreated chondrocytes was observed in coculture with H-EVs and the effect was smaller (average 4% increase) in coculture with OA-EVs however the differences failed to reach statistical significance (Figure 3(b)). A trend for lower-level upregulation of *SOX9* by OA-EVs compared to H-EVs, as well as of transcripts for mature cartilage ECM proteins *COL2* and *ACAN* was evident following longer-term culture (Figure 3(c)). Higher *COL2*/*COL1* ratio indicating chondrogenic lineage commitment displayed the same trend, unlike the expression of cartilage catabolic molecules where no specific trends were found (both short- and long-term) (Figure 3(c)).

### 3.4. miRNA Profiling

**Figure 4:**
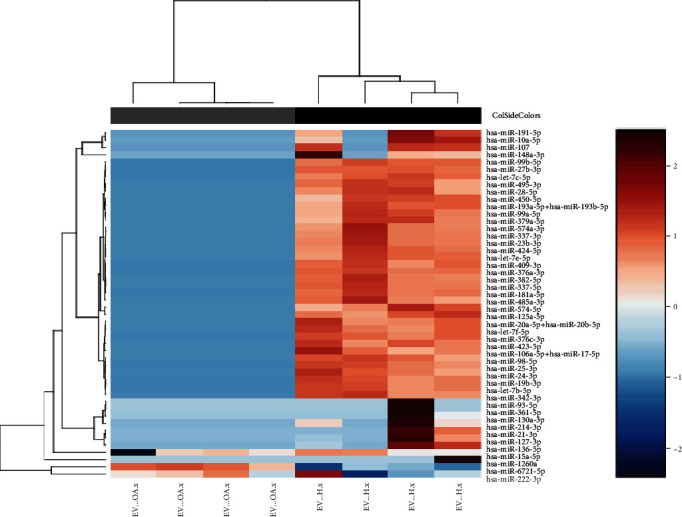
OA-EV and H-EV miRNA expression profiling using NanoString technology. Heatmap showing unsupervised hierarchical clustering of significantly differentially expressed microRNAs (*p* < 0.05, *n* = 48), based on normalized digital expression counts in OA-EVs vs. H-EVs. Each column represents an individual sample. Relative expression changes are indicated by the colour key (red: high; blue: low). OA-EVs are depicted by grey shading, while H-EVs are depicted by black shading.

Albeit not being statistically significant, average of 1.5-fold differences in chondrocytes' chondrogenesis gene expression (*SOX9*, *COL2* and *ACAN* expression at 21 days), indicated potential differences in microRNA cargo between H-EVs and OA-EVs. microRNA expression profiling was next performed on H-EVs and OA-EVs (*n* = 4 cultures each) using NanoString technology (*n* = 799 microRNA). Expression of 590 mature microRNAs was detected across all samples after correcting for background (Supplementary Data 1). Healthy and OA-EVs showed distinct microRNA expression profiles, as demonstrated using unsupervised hierarchical clustering analysis (Figure 4). There were 75 miRNAs that were significantly differentially expressed between H-EVs and OA-EVs, of which 48 retained significance after FDR correction. A total of 47 were upregulated in healthy EVs and 1 in OA-EVs (fold change (FC) range = −12.3 − 26.67, *p* value range = 0.002-0.037) (Figure 4).

The 48 DE-expressed microRNAs were predicted to target KEGG pathways [50] using mirPath [45], with key implications in chondrogenesis or stem cells, including extracellular matrix interaction (56 genes, 36 microRNAs, *p* < 0.001), N-glycan biosynthesis (30 genes, 29 microRNA, *p* < 0.001), focal adhesion (133 genes, 43 microRNAs, *p* < 0.001) and signalling pathways regulating pluripotency of stem cells (94 genes, 45 microRNAs, *p* < 0.001). Predicted gene targets identified with ‘very high' score class (top 1%) via the microRNA Data Integration Portal (mirDIP) were filtered for duplicates, resulting in 10,755 unique gene targets. Reactome pathways implicated by the target genes included cell-cell communication (95/133 genes), transcriptional regulation of pluripotent stem cells (38/45 genes), extracellular matrix organisation (207/329 genes), transport of small molecules (483/967 genes), and vesicle-mediated transport (510/824 genes). miRWalk also predicted target map to signalling pathways “regulating pluripotency of stem cells” pathway.

In addition to microRNAs that were significantly differentially expressed between OA-EVs and H-EVs, we also assessed the top 20 most highly expressed microRNAs in each population, as well as those that were commonly highly expressed in both OA and H-EVs. 17 microRNA were expressed at a high level in both populations, while miR-6721-5p, miR-579-3p and miR-199a-5p were uniquely highly expressed in OA-MSC-EVs, and miR-145-5p, miR-126-3p and miR-15b-5p were uniquely highly expressed in control healthy MSC-EVs (Table 3).

**Figure 5:**
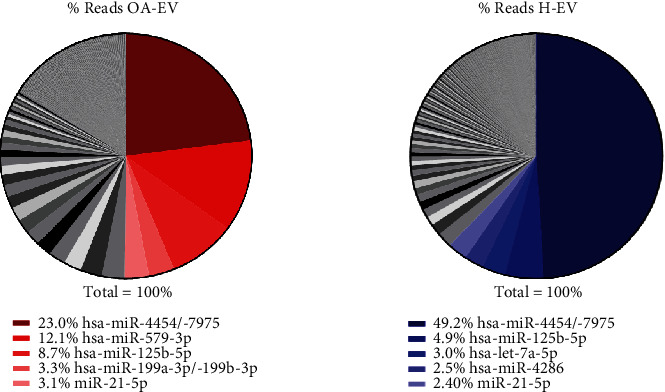
OA-EV and H-EV miRNA profiling analysis. Diagrams showing the percentages of reads from the top 5 most highly expressed miRNAs. H-EVs: EVs from control healthy MSCs; OA-EVs: EVs from OA-MSCs.

The top 5 most highly expressed microRNAs in each population comprised over 50% of all microRNA reads (OA − EVs = 50.2%, H − EVs = 62.0%) (Figure 5), of which miR-4454/-7975, miR-125b-5p, and miR-21-5p were commonly highly expressed.

**Figure 6:**
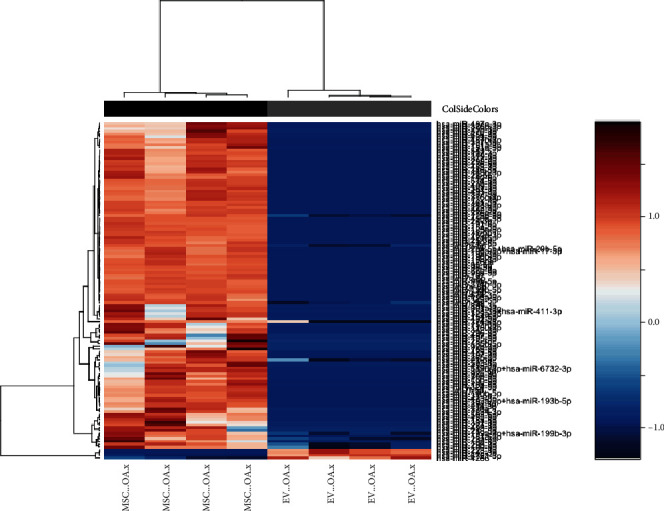
miRNA profiling of OA-EVs and their parental MSCs using NanoString technology. Heatmap showing unsupervised hierarchical clustering of significantly differentially expressed microRNAs (*p* < 0.05, *n* = 124), based on normalized digital expression counts in OA-MSC-EVs vs. OA-MSCs. Each column represents an individual sample. Relative expression changes are indicated by the colour key (red: high; blue: low). OA-MSC-EVs are depicted by grey shading, while MSC are depicted by black shading.

When we compared miRNAs in OA-EVs with their parental MSCs (Supplementary Data 2), we found that they clustered separately (Figure 6). There were 130 miRNAs that were significantly differentially expressed between OA-EVs and parental OA-MSCs, of which 124 retained significance after FDR correction (Figure 6). A total of 120 were upregulated in healthy EVs and 4 in OA-EVs (fold change (FC) range = −75.95 − 68.62, *p* value range ≤ 0.001-0.049) (Figure 4).

For KEGG pathway analysis [50], 100 miRNA with higher FC out of the 124 DE expressed microRNAs were analyzed using mirPath [45]. The same key implications in chondrogenesis or stem cells were found as above, with the exception of pathways regulating pluripotency of stem cells: extracellular matrix receptor interaction (71 genes, 77 microRNAs, *p* < 0.001), N-glycan biosynthesis (44 genes, 58 microRNA, *p* < 0.001) and focal adhesion (177 genes, 82 microRNAs, *p* < 0.001). Other pathways observed were proteoglycans in cancer (180 genes, 80 microRNAs, *p* < 0.001), fatty acid metabolism (42 genes, 66 mircroRNAs, *p* < 0.001) or cell cycle (111 genes, 77 microRNAs, *p* < 0.001). Predicted gene targets with ‘very high' score class (top 1%) via the microRNA Data Integration Portal (mirDIP) were identified, resulting in 100,714 unique gene targets. Reactome pathways implicated by the target genes included MECP2 regulated neuronal receptors and channels (32/32), transcription factor forkhead box O (FOXO)-mediated transcription (85/110 genes), TP53 regulated transcription of genes involved in G1 cell cycle arrest (20/20), transcriptional regulation by RUNX family transcription factor 3 (RUNX3) (87/118 genes), extracellular matrix organization (249/329 genes), programmed cell death (170/238 genes), and vesicle-mediated transport (552/824 genes). miRWalk predicted target map to signalling pathways related to cartilage and osteoarthritis as “glycolysis_gluconeogenesis”, “fatty acid metabolism”, “glycosaminoglycan biosynthesis”, “autopaghy”, “FOXO signaling pathway”, “calcium signaling pathway”, “osteoclast differentiation”, and other pathways like “endocytosis” or “cell cycle”.

Assessing the top 20 most highly expressed microRNAs, 14 microRNAs were expressed at a high level in both parental OA-MSCs and their EVs, while 6 miRNAs were uniquely highly expressed in OA-MSC-EVs and other 6 were uniquely highly expressed in parental MSCs (Table 4).

## 4. Discussion

Previous studies on EVs in OA have almost exclusively focused on the synovial fluid (SF) EVs, which mediate the cross-talk between the damaged cartilage and the inflamed synovium [51, 52] and have shown their considerable influence on chondrocyte catabolism, senescence, and death [53–57]. In contrast, the cross-talk between subchondral bone (SB) cells and chondrocytes in OA is comparatively less explored, despite the fact that SB pathology is implicated in all stages of OA progression [13, 58, 59]. Furthermore, in previous studies of SF-resident EVs, their tissues of origin, as well as the cells of origin, remained unknown. In contrast, our study is uniquely focused on the cross-talk between cartilage and SB in OA and investigated EV production from a specific type of SB cells, SB MSCs, which are directly implicated in SB OA pathology and cartilage destruction “from below” [60–62]. Although SB MSCs from healthy amputees would be a better control than the MSCs from bone marrow used in this work, these samples are difficult to access while MSCs from amputated knees from diabetics could carry underlying negative effects such as described previously [63–65].

In this work, when comparing EV size and yields (particle/cell) released from OA and control healthy MSCs, no differences were found, similar to results previously described for healthy and OA-EVs from SF [55, 57]. Previous works on SF-EVs have presented slightly lower EV sizes [53–55], which could be explained by different methods used for EV isolation in these studies: using ultracentrifugation combined with precipitation [54, 55] or using immunoaffinity [53]. EVs obtained from ultracentrifugation alone, as was in our study, are known to be less purified due to the overlap in sizes between EVs and microvesicles [25], however ultracentrifugation is less harmful for EVs [66].

One of the principal aims of this study was to identify specific sets of miRNAs in the OA-EVs derived from SB-MSCs. Depending on the cell of origin, EVs (including exosomes) can contain many constituents of a cell including DNA, RNA, lipids, metabolites, and cytosolic and cell- surface proteins [67]. We have identified several miRNAs that in our experimental conditions were packaged into their EVs and may be characteristic of OA-EVs from these cells. Many previous studies compared miRNA expression in MSC-EVs and their parental MSCs [68–72], but these studies were all focused on healthy MSCs. Our study has identified 14 miRNAs that were highly expressed in both OA-MSC-EVs and their parental SB MSCs. These included miR-125b, which has been previously shown to regulate the expression of matrix-degrading enzymes in human chondrocytes [73–75] as well as regulate MSC osteogenesis [76], and miR-199a involved in repression of chondrogenesis [77], regulation of chondrocyte ageing and cartilage metabolism [78], and highly expressed in OA SF EVs [57]. Previous studies including ours [18, 61, 79] have described gene expression profiles of OA SB MSCs as predisposed towards bone formation and cartilage extracellular matrix regulation. The current study complements these findings by showing that highly expressed miRNA in these MSCs, including those packaged in their EVs, may be regulating cartilage homeostasis in OA. Although further functional analysis would be necessary to confirm that these miRNAs contained in the EVs may produce this chondrogenic effect.

Furthermore, the present study has identified four miRNAs that are higher-expressed in OA-MSC-EVs compared to parental MSCs and may be involved in regulating the viability and the metabolic status of OA chondrocytes. Of a particular interest are miR-142-3p previously shown to be selectively packaged into EVs [80] and miR-223-3p that have been both shown to inhibit cell apoptosis and inflammation in OA chondrocytes [81, 82], as well as miR-630 that has been earlier documented to regulate chondrogenic lineage commitment [83]. In our previous work we have used qPCR and validated the enrichment of miR-142-3p and miR-223-3p in H-EVs coming from MSCs [41]. The role of miR-630 and other miRNAs has not yet been explored in OA and is awaiting further validation.

Pathway analysis has indicated that OA-EVs and/or OA-MSCs expresses miRNAs implicated in chondrogenesis, stem cells, or other pathways related to cartilage and OA. Interestingly, “fatty acid metabolism” points out to the already known role of fatty acids in OA [84]. Also, FOXO transcription factors have been described as chondroprotectors and regulating autophagy and inflammation [85]. Altogether, these data suggest that despite their altered gene expression and predisposition to osteogenesis in OA, SB MSCs produce miRNAs and release EVs that contain miRNAs involved in positive regulation of chondrocyte viability and differentiation.

MSC-EVs from various human tissues including bone marrow (BM) and adipose tissue are beginning to be used as a therapy for OA [52, 86]. In this context, it was interesting to compare the miRNA cargo of OA-EVs with H-EVs obtained from BM aspirates from healthy individuals. Amongst top highly expressed miRNAs, 17 were shared in both types of EVs (Table 3), 13 of which were also abundant in OA-MSCs (Table 4). Common abundant miRNAs included miR-16-5p previously used as an endogenous reference gene for the normalization of urinary exosomal miRNA expression [87], and some let family members previously described amongst the top 10 most abundant miRNAs in humans' samples [88, 89]. Common miRNAs between OA-EVs and H-EVs that were not present in OA-MSCs included previously noted miR-142-3p and miR-630 [86] that have been described as “cartilage-protective.” Also miR-29a-3p was described as protective for cartilage [86]. Healthy EVs higher-expressed miR-145-5p (uniquely highly expressed in H-EVs compared to OA-EVs) described as having a dual role in OA [90], while OA-EVs higher-expressed hsa-miR-199a-5p involved in chondrogenesis regulation and bone formation [91], as well as highly expressed in OA SF EVs [57] and in plasma of early OA patients [92]. Our *in vitro* data have revealed that both H-EVs and OA-EVs marginally but significantly improved the viability of OA chondrocytes, with no difference to each other, which could be a result of consorted activity of multiple miRNAs.

Recent *in vitro* studies on MSC-EVs in OA have used inflammatory stimulation of chondrocytes before coculturing with H-EVs [93–96], and they obtained a reduction in the expression of chondrocyte inflammatory markers. It could be possible that this stimulation made the EV-effect on chondrocytes stronger than observed in our study. In our study, we aimed to reflect more closely the in vivo conditions of chondrocytes based on our previous findings that OA chondrocytes grown in the same conditions already present the signs of inflammation, evident by high-level expression of matrix-degrading enzymes and pro-inflammatory cytokines [18].

Even though our data on MSC-EV (both OA- and H-EVs) effects on chondrocyte gene expression are limited, they point towards a worse induction of chondrogenic gene expression by OA-EVs compared to H-EVs. The lack of statistical significance could be explained by donor-to-donor variation in both chondrocytes and EVs preparations that we have used. To minimise donor effects and generate larger EV batches for use in multiple experiments, future studies could utilise EVs [54] or chondrocytes [49] which are pooled from several donors.

Smaller than expected effects of EVs on chondrocyte gene expression could be also explained by the 3D assay conditions we used. Mortati and colleagues [49] added MSC-EVs to 4-week cultured-chondrocyte pellets and showed that EVs could diffuse into the matrix. Differently from their study, we mixed the EVs with the chondrocytes before the 3D pellet formation as investigated their effect at different time points of chondrogenesis. Also, no chondrogenic inducers such as TGF*β*1 [49] were added to the media in our study because these strong inducers could “mask” or conflict with potentially smaller effects from MSC-EVs. In support of our assay design, previous studies have documented the presence of TGF*β*1 in MSC-EVs [97], which could explain long-term, enhanced chondrogenic gene expression with the addition of H-EVs in our experiments. While some studies report on MSC-EV enhancement of chondrocyte collagen II expression in 2D formats [94], 3D pellets [48], cells encapsulated in cartilage-mimicking hydrogels [49] are likely to provide conditions more representative to EV traffic *in vivo*. Specific to OA osteochondral pathology, such constructs should take into the account intercellular distances between SB MSCs and chondrocytes, which can be established from histological studies [6, 18], as well as a known heterogeneity of chondrocyte subpopulations in OA [98, 99], which is difficult to re-capitulate in *in vitro* systems.

In summary, OA-EVs were capable to enhance the viability of OA chondrocytes; also, the expression of chondrogenic genes was enhanced in the chondrocytes by the EVs from OA-MSCs. However, both effects were less evident than the ones shown for H-EVs. This variation could be partially explained by the differential expression of miRNAs found in the cargo of H-EVs and OA-EVs.

## 5. Conclusions

The present study reports on the production, characterization and miRNA profile of EVs from SB MSCs in OA. For the first time, it shows that SB MSCs in OA are capable of producing EVs that support chondrocyte viability and chondrogenic gene expression, although future studies would be needed to confirm it using functional assays for chondrogenesis. This supports the notion that modulation of SB MSCs represents a valid strategy for endogenous cartilage regeneration in OA. This may be achieved via enhancement of their trophic activity and EV production in addition to triggering their chondrogenic differentiation, thus representing a new potential tool for cartilage regeneration.

## Figures and Tables

**Table 1:** Taqman assays used for qPCR on MSC-EV (both OA- and H-EVs) treated chondrocytes.

Gene name	Gene symbol	Probe/assay ID
SRY (sex determining region Y)-box 9	*SOX9*	Hs00165814_m1
Collagen type II alpha 1 chain	*COL2A1*	Hs00264051_m1
Aggrecan	*ACAN*	Hs04982230_s1
Collagen type I alpha 2 chain	*COL1*	Hs01028969_m1
Matrix metallopeptidase 13	*MMP13*	Hs00942586_m1
A disintegrin and metalloproteinase with thrombospondin motifs 4	*ADAMTS4*	Hs00192708_m1
A disintegrin and metalloproteinase with thrombospondin motifs 5	*ADAMTS5*	Hs01095518_m1
Glyceraldehyde 3-phosphate dehydrogenase	*GAPDH*	Hs02758991_g1

**Table 2:** Summary of EV characteristics isolated from OA and healthy MSCs: particle size, concentration of particles, and the number of cells from which EVs were obtained.

Sample	Particle size			Concentration of particles		Particles/cell∗
	Mean (nm)	SD (nm)	Mode (nm)	Particles/ml∗	Particles/frame∗	
OA-EV1	161.6	69.3	169.8	1.19 × 10^11^ ± 1.64 × 10^10^	12.1 ± 1.7	692 ± 95
OA-EV2	172.2	75.0	121.1	1.30 × 10^11^ ± 1.69 × 10^10^	13.2 ± 1.7	531 ± 69
OA-EV3	177.2	87.4	158.4	1.15 × 10^11^ ± 1.07 × 10^10^	11.7 ± 1.1	622 ± 58
OA-EV4	160.0	71.6	133.7	1.35 × 10^11^ ± 3.20 × 10^10^	13.7 ± 3.2	433 ± 103
OA-EV5	172.0	71.1	107.9	1.92 × 10^11^ ± 2.75 × 10^10^	29.2 ± 4.2	897 ± 128
H-EV1	184.1	89.4	132.9	2.36 × 10^11^ ± 2.53 × 10^10^	77.4 ± 8.1	3670 ± 393
H-EV2	152.3	67.4	110.2	7.43 × 10^10^ ± 4.76 × 10^9^	61.7 ± 4.0	1265 ± 81
H-EV3	125.2	52.9	100.7	5.58 × 10^10^ ± 2.9 × 10^9^	27.2 ± 1.5	1240 ± 64
H-EV4	154.8	63.6	107.9	9.41 × 10^10^ ± 1.11 × 10^10^	46.7 ± 5.7	752 ± 88
H-EV5	129.6	55.3	81.3	5.76 × 10^10^ ± 3.32 × 10^9^	21.6 ± 1.4	694 ± 40
H-EV6	152.1	79.7	98.4	1.53 × 10^11^ ± 3.59 × 10^9^	77.7 ± 2.2	3035 ± 71

∗Data shows mean ± SD from measurements of 3 different frames within the Nanosight.

**Table 3:** microRNAs highly expressed in OA-EVs and control H-EVs.

Names	Total	microRNAs
Shared OA-EVs & H-EVs	17	hsa-miR-142-3p
		hsa-miR-199a-3p + hsa-miR-199b-3p
		hsa-miR-4286
		hsa-let-7a-5p
		hsa-miR-16-5p
		hsa-miR-21-5p
		hsa-miR-29a-3p
		hsa-miR-29b-3p
		hsa-let-7i-5p
		hsa-miR-4454+ hsa-miR-7975
		hsa-miR-630
		hsa-let-7b-5p
		hsa-miR-221-3p
		hsa-miR-23a-3p
		hsa-miR-100-5p
		hsa-miR-125b-5p
		hsa-miR-223-3p

OA-EVs	3	hsa-miR-6721-5p
		hsa-miR-579-3p
		hsa-miR-199a-5p

H-EVs	3	hsa-miR-145-5p
		hsa-miR-126-3p
		hsa-miR-15b-5p

**Table 4:** microRNAs highly expressed in OA-MSCs and OA-EVs.

Names	Total	microRNAs
Shared OA-EVs & OA-MSCs	14	hsa-miR-199a-3p+ hsa-miR-199b-3p
		hsa-let-7a-5p
		hsa-miR-16-5p
		hsa-miR-21-5p
		hsa-miR-29a-3p
		hsa-miR-29b-3p
		hsa-let-7i-5p
		hsa-miR-4454+ hsa-miR-7975
		hsa-let-7b-5p
		hsa-miR-221-3p
		hsa-miR-199a-5p
		hsa-miR-23a-3p
		hsa-miR-100-5p
		hsa-miR-125b-5p

OA-EVs	6	hsa-miR-142-3p
		hsa-miR-4286
		hsa-miR-6721-5p
		hsa-miR-630
		hsa-miR-579-3p
		hsa-miR-223-3p

OA-MSCs	6	hsa-miR-199b-5p
		hsa-miR-15a-5p
		hsa-let-7c-5p
		hsa-miR-99a-5p
		hsa-miR-15b-5p
		hsa-let7g-5p

## Data Availability

miRNA profiling data are attached as supplementary files.

## Supplementary Materials

Supplementary 1 Supplementary Figure 1: characterization of MSCs from subchondral bone (SB) of OA patients (OA-MSCs). (A) Trilineage differentiation of OA-MSCs following induction and staining with Oil Red O (day 21), Alkaline phosphatase (day 14) and Toluidine Blue (day 21), for adipogenesis, osteogenesis and chondrogenesis, respectively. (B) Phenotypic profile of OA-MSCs showing the % expression of CD73, CD90, CD105, hematopoietic-lineage markers (CD45, CD34, CD19, CD14) and HLA-DR measured by flow cytometry. Original magnification x200 for adipo- and osteo- genesis and x40 for chondrogenesis.

Supplementary 2 Supplementary Figure 2: characterization of MSCs from healthy controls (H-MSCs). (A) Trilineage differentiation of H-MSCs following induction and staining with Oil Red O (day 21), Alkaline phosphatase/Von Kossa (day 14) and Alcian Blue (day 21) for adipogenesis, osteogenesis and chondrogenesis, respectively. (B) Phenotypic profile of H-MSCs showing the % expression of CD73, CD90, CD105, hematopoietic-lineage markers (CD45, CD34, CD19, CD14) and HLA-DR measured by flow cytometry. Scale bar 200 *μ*m for adipo- and osteo- genesis and 500 *μ*m for chondrogenesis.

Supplementary 3 Supplementary Data 1: analysis of miRNAs in OA-EVs compared with H-EVs, showing the differential expression, with and without FDR correction, fold change (FC) and *p* value.

Supplementary 4 Supplementary Data 2: analysis of miRNAs in OA-EVs compared with their parental MSCs, showing the differential expression, with and without FDR correction, fold change (FC) and *p* value.
