# Supplementary figures and images for: Characterization and miRNA Profiling of Extracellular Vesicles from Human Osteoarthritic Subchondral Bone Multipotential Stromal Cells (MSCs)
Source: Stem Cells Int. 2021 Oct 9;2021:7232773. doi: 10.1155/2021/7232773 (PMC8520657; doi:10.1155/2021/7232773)

## Slide 1
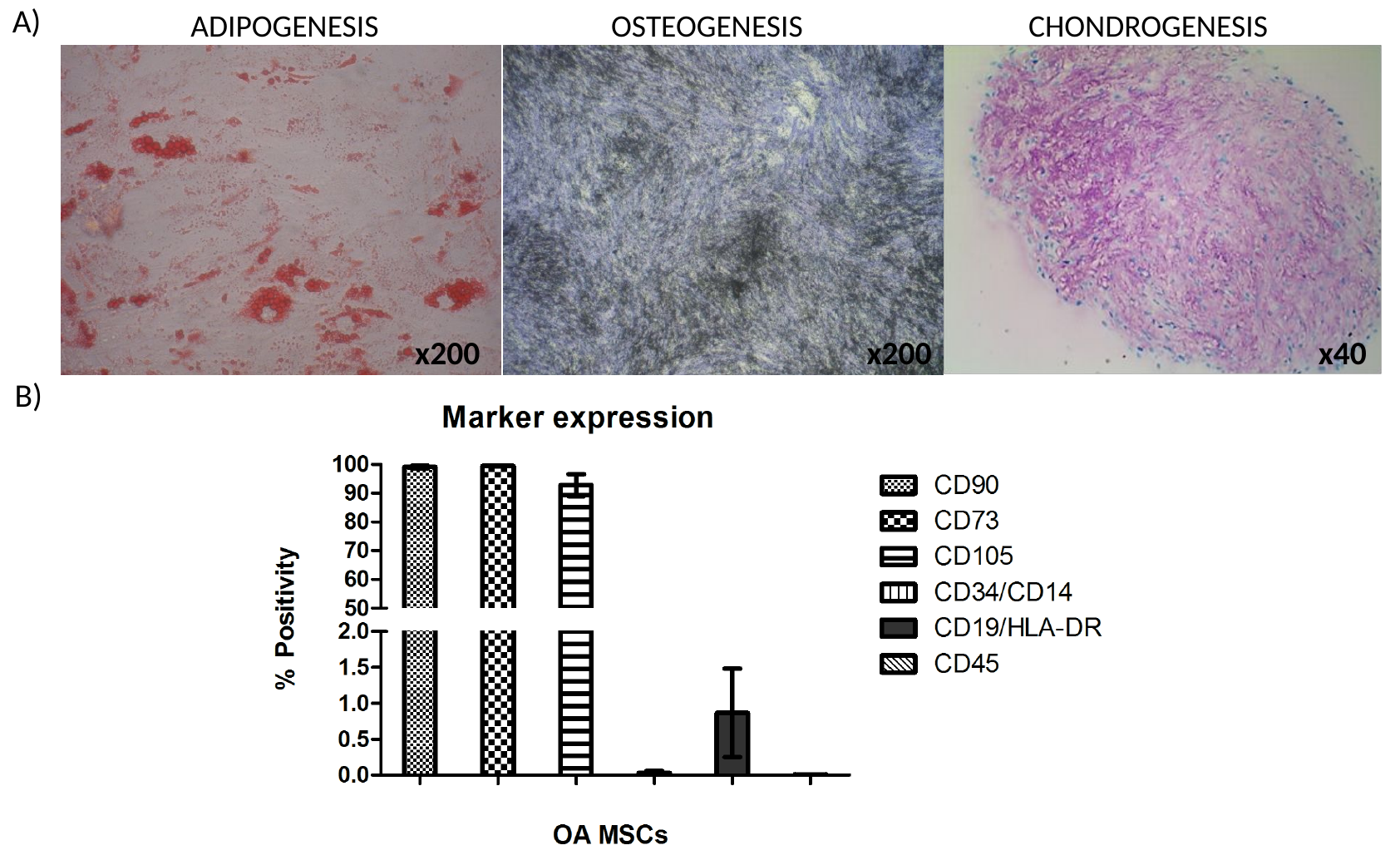

ADIPOGENESIS OSTEOGENESIS CHONDROGENESIS
A)
x200
x200
x40
B)

Supplement: Supplementary 1 — Supplementary Figure 1: characterization of MSCs from subchondral bone (SB) of OA patients (OA-MSCs). (A) Trilineage differentiation of OA-MSCs following induction and staining with Oil Red O (day 21), Alkaline phosphatase (day 14) and Toluidine Blue (day 21), for adipogenesis, osteogenesis and chondrogenesis, respectively. (B) Phenotypic profile of OA-MSCs showing the % expression of CD73, CD90, CD105, hematopoietic-lineage markers (CD45, CD34, CD19, CD14) and HLA-DR measured by flow cytometry. Original magnification x200 for adipo- and osteo- genesis and x40 for chondrogenesis. [file 7232773.f1.pptx]

## Slide 1
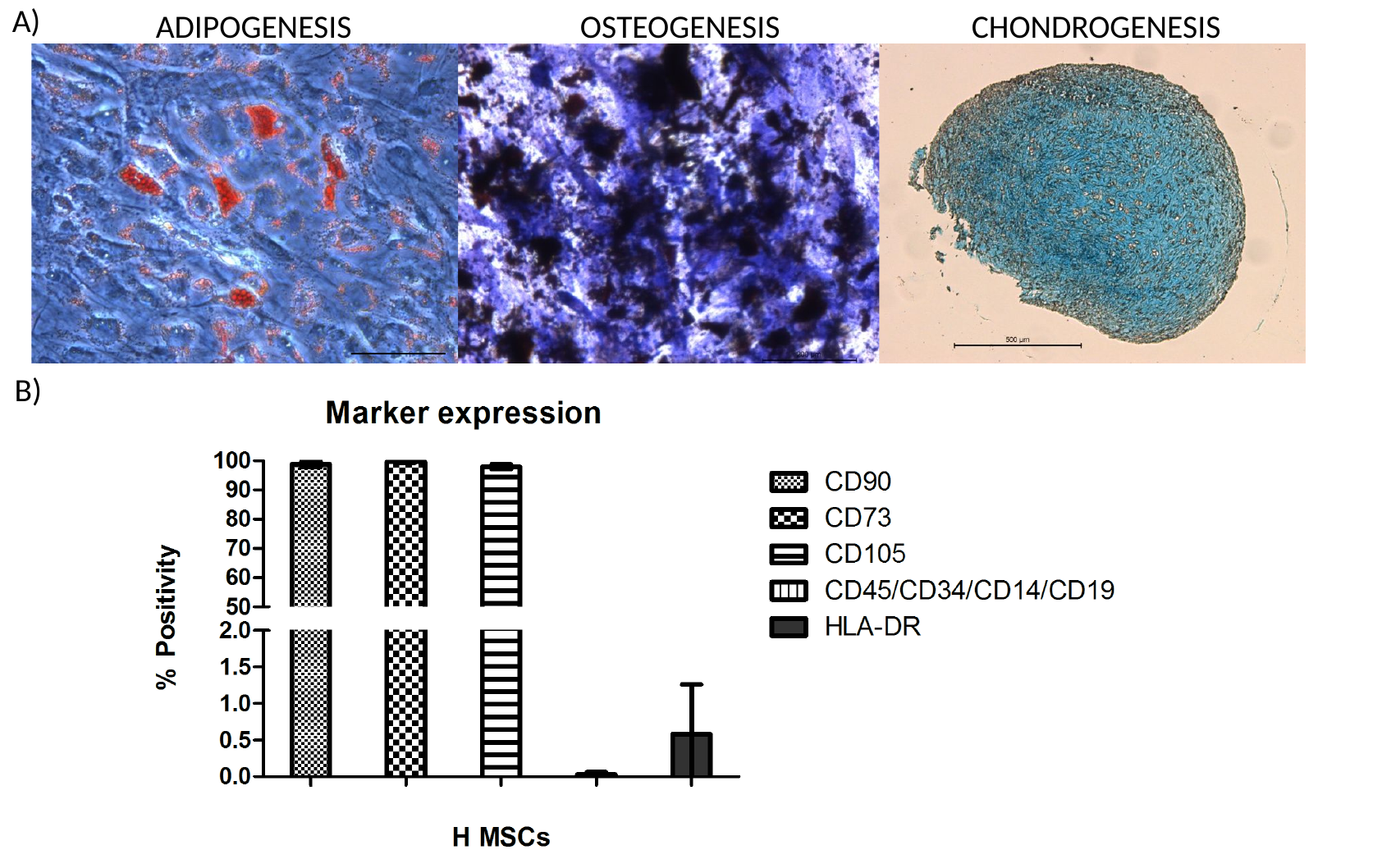

A)
 ADIPOGENESIS OSTEOGENESIS CHONDROGENESIS
B)

Supplement: Supplementary 2 — Supplementary Figure 2: characterization of MSCs from healthy controls (H-MSCs). (A) Trilineage differentiation of H-MSCs following induction and staining with Oil Red O (day 21), Alkaline phosphatase/Von Kossa (day 14) and Alcian Blue (day 21) for adipogenesis, osteogenesis and chondrogenesis, respectively. (B) Phenotypic profile of H-MSCs showing the % expression of CD73, CD90, CD105, hematopoietic-lineage markers (CD45, CD34, CD19, CD14) and HLA-DR measured by flow cytometry. Scale bar 200 μm for adipo- and osteo- genesis and 500 μm for chondrogenesis. [file 7232773.f2.pptx]
